# Risk Factors for Delayed Neurocognitive Recovery According to Brain Biomarkers and Cerebral Blood Flow Velocity

**DOI:** 10.3390/medicina56060288

**Published:** 2020-06-11

**Authors:** Rasa Bukauskienė, Edmundas Širvinskas, Tadas Lenkutis, Rimantas Benetis, Rasa Steponavičiūtė

**Affiliations:** 1 Department of Cardiothoracic and Vascular Surgery, Lithuanian University of Health Sciences, 50161 Kaunas, Lithuania; edmundas.sirvinskas@lsmuni.lt (E.Š.); tadas.lenkutis@lsmuni.lt (T.L.); rimantas.benetis@lsmuni.lt (R.B.); 2 Department of Laboratory Medicine, Lithuanian University of Health Sciences, 50161 Kaunas, Lithuania; rasa.steponaviciute@lsmuni.lt

**Keywords:** cardiac surgery, delayed neurocognitive recovery, bypass, cardiac anesthesia, transcranial Doppler ultrasound

## Abstract

*Background and Objectives:* The aim of this study is to identify risk factors for the development of delayed neurocognitive recovery (dNCR). *Materials and Methods:* 140 patients underwent neurocognitive evaluations (Adenbrooke, MoCa, trial making, and CAM test) and middle cerebral artery (MCA) blood flow velocity (BFV) measurements, one day before cardiac surgery. BFV was re-evaluated after anesthesia induction, before the beginning, middle, end, and after cardiopulmonary bypass (CPB) and postsurgery. To measure glial fibrillary acidic protein (GFAP) and neurofilament heavy chain (Nf-H), blood samples were collected after anesthesia induction, 24 and 48 h after the surgery. Neurocognitive evaluation was repeated 7–10 days after surgery. According to the results, patients were divided into two groups: with dNCR (dNCR group) and without dNCR (non-dNCR group). *Results:* 101 patients completed participation in this research. GFAP increased in both the non-dNCR group (*p* < 0.01) and in the dNCR group (*p* < 0.01), but there was no difference between the groups (after 24 h, *p* 0.342; after 48 h, *p* 0.273). Nf-H increased in both groups (*p* < 0.01), but there was no difference between them (after 24 h, *p* = 0.240; after 48 h, *p* = 0.597). MCA BFV was significantly lower in the dNCR group during the bypass (37.13 cm/s SD 7.70 versus 43.40 cm/s SD 9.56; *p* = 0.001) and after surgery (40.54 cm/s SD 11.21 versus 47.6 cm/s SD 12.01; *p* = 0.003). Results of neurocognitive tests correlated with CO_2_ concentration (Pearson’s r 0.40, *p* < 0.01), hematocrit (r 0.42, *p* < 0.01), MCA BFV during bypass (r 0.41, *p* < 0.01), and age (r −0.533, *p* < 0.01). The probability of developing dNCR increases 1.21 times with every one year of increased age (*p* < 0.01). The probability of developing dNCR increases 1.07 times with a decrease of BFV within 1 cm/s during bypass (*p* = 0.02). *Conclusion*: Risk factors contributing to dNCR among the tested patients were older age and middle cerebral artery blood flow velocity decrease during bypass.

## 1. Introduction

Delayed neurocognitive recovery (dNCR) remains a frequent condition after surgery and is characterized as impairment of memory, attention, and concentration [1]. The frequency of dNCR is described between 30–80% [2] and depends on the type of surgery. The dNCR rate is higher for patients receiving cardiac surgery with cardiopulmonary bypass (CPB) [1]. dNCR is associated with longer hospitalization, poor cognitive and functional recovery, as well as increased mortality [2].

The etiology of dNCR is associated with genetics, inflammatory neurotransmitters, stress, anesthesia, and other factors [1,2]. There are many known risk factors for the development of dNCR. Risk factors are related to the patient (e.g., age, comorbidities, cognitive function before the surgery), surgery (e.g., CPB and cross-clamping duration, bleeding), anesthesia (anesthetics, hypotension, ventilation), and postoperative factors (duration of ventilation, bleeding) [3]. Identifying the most important risk factors may help to reduce the incidence of dNCR, and interventions could be conducted to minimize cognitive impairments postoperation [3].

Many researchers have identified that neuropsychological complications emerge from insufficient cerebral perfusion [4,5,6,7]. Transcranial Doppler sonography (TCD) could provide information about middle cerebral artery (MCA) blood flow velocity (BFV) during surgery to detect hypoperfusion episodes and cerebral embolism. Additionally, TCD has the advantage of being a noninvasive, repeatable, and safe method. TCD may be helpful in detecting MCA BFV impact on dNCR development and clarifying risk factors for cerebral hypoperfusion.

Early detection of blood-based brain biomarkers could correlate with neurological injury [8]. Neuroinflammation is one of the most important factors at the beginning of brain injury and dNCR [9,10]. Neurofilaments are protein polymers found in the cytoplasm of neurons and could be specific biomarkers to detect neuroaxonal injury [11]. It is a useful marker for amyotrophic lateral sclerosis, multiple sclerosis, Huntington’s disease, Parkinson’s disease, and sepsis-associated encephalopathy [11]. The value of neurofilaments in cardiac surgery has not been sufficiently studied and has just started to be explored. The glial fibrillary acidic protein (GFAP) is a monomeric filament protein found in the astroglial skeleton and is a specific marker of brain damage [12]. Elevation in plasma GFAP levels has been reported in adults with traumatic brain injury, stroke, or after cardiac arrest [5,8,13]. Monitoring plasma GFAP levels are often used in research after cardiac and noncardiac surgery to help identify brain injury and dNCR.

The aim of this study is to determine the most important factors in dNCR development based on neurocognitive evaluation and brain biomarkers neurofilament heavy chain (Nf-H) and GFAP. 

## 2. Materials and Methods

**Figure 1:**
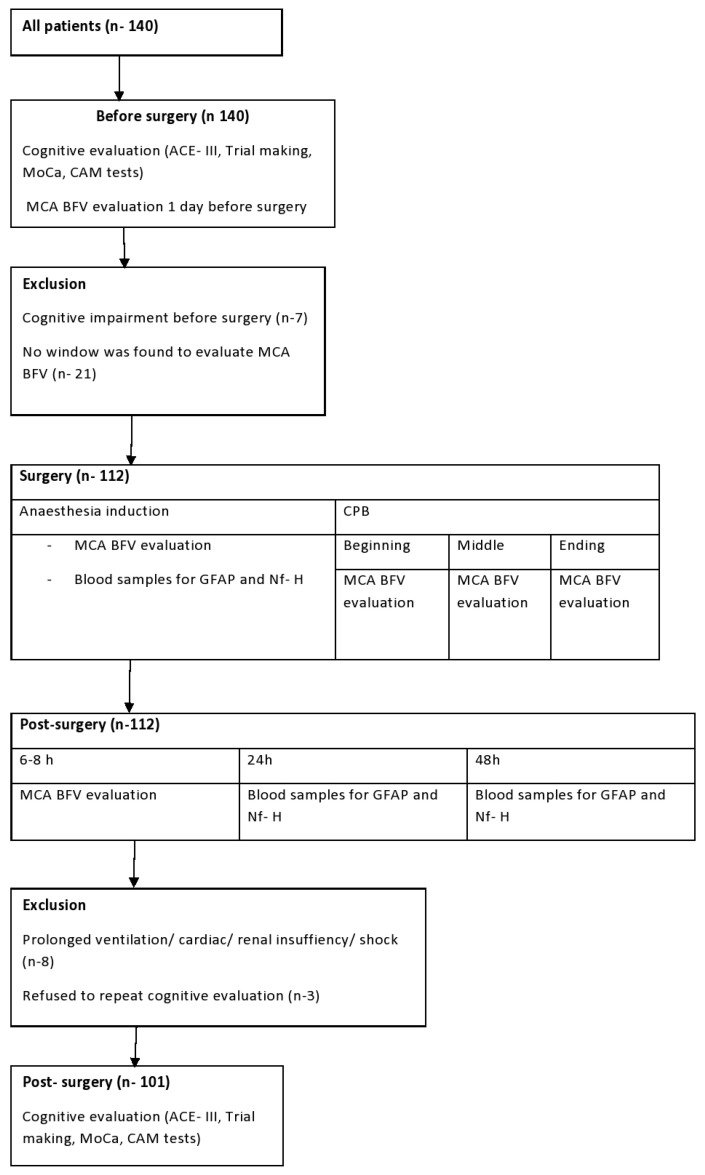
Study design and patient flow. MCA—middle cerebral artery; BFV—blood flow velocity; CPB—cardiopulmonary bypass.

This prospective observational study was conducted for 140 patients undergoing elective heart surgery at the Department of Cardiothoracic and Vascular Surgery in the Hospital of Lithuanian University of Health Sciences Kauno Klinikos from April 2018 to April 2019. Permission was obtained from, and the research was approved by the Kaunas Regional Bioethics Committee (Nr BE-2-3) and the Institutional Review Board (NCT03641833). All patients signed a “participation in research” form. Inclusion criteria were elective heart surgery with CPB and age > 18 years. Exclusion criteria were patients suffering from diseases causing cognitive dysfunction (stroke, epilepsy) or using agents affecting the central nervous system, multiple organ dysfunction syndrome, deficiency, emergency or re-surgery, carotid artery atherosclerosis with a reduction in artery diameter of 50% or more, and patient’s disagreement. The study design is shown in Figure 1. 

### 2.1. Cognitive Evaluation

Neurocognitive evaluation tests were performed one day before surgery and 7–10 days postsurgery before leaving the hospital. They were chosen to determine many different domains and included:

1. The Adenbrooke test (ACE-III) (adopted in the Lithuanian language by R. Margeviciute, A. Bagdonas, K. Butkus, J. Kuzmickiene, A. Vaitkevicius, G. F. Kaubrys, T. H. Bak in 2013 September is composed of five cognitive domains: attention, memory, language, verbal fluency, and visuospatial abilities. It is sensitive to mild cognitive impairment.
2. Trial making test, part A—provides information about visual attention and processing speed. Montreal Cognitive Assessment (MoCa)—validated to detect cognitive impairment and is often used instead of the Mini mental state examination (MMSE) test [14].
3. Confusion Assessment Method (CAM)—used to identify delirium.

### 2.2. Perioperative Period

All patients received premedication of 1–2 mg oral clonazepam and the half dose of the used dose of metoprolol. 

Patients received general anesthesia. Induction was performed with fentanyl 1–2 μg/kg i.v.; propofol 2–2.5 mg/kg i.v.; rocuronium 0.5–0.7 mg/kg. Lungs were ventilated with a supply of oxygen/air 50–60% after intubation. Anesthesia was maintained with sevoflurane (MAC 1–2%) and fentanyl (10–12 μg/kg) for analgesia. Middle sternotomy section and standard technique for anastomosis formation and valve surgery were performed for all the patients. After systemic heparinization and cannulation, CPB was established using a roller pump, “Stockert III”, and hollow-fiber membranous oxygenator “Compactflo D703” (Dideco, Mirandola, Italy). The CPB circuit was primed with 1200–1500 mL of Ringer’s acetate crystalloid solution and 10,000 IU of heparin. After full CPM support, pump flow was maintained at a cardiac index of 2.4 L/min/m [2]. Cardiac arrest was achieved with cold hypercalcemic St. Thomas according to clinical protocol.

Standard monitoring of vital signs consisted of an electrocardiogram, heart rate, invasive arterial blood pressure, pulse oximetry, esophageal and body core temperature, central venous pressure, and urine output. Bispectral index (BIS) was maintained 40–60 for adequate depth of anesthesia. Mean arterial pressure was maintained between 50 and 75 mmHg by infusion of intravenous fluids and administration of sympathomimetics if needed. Normothermia was maintained during surgery. 

All patients were transferred to the intensive care unit (ICU) after surgery and treated according to ICU protocols.

### 2.3. TCD Technique

The TCD technique was performed with a Sonara transcranial Doppler device with 2 MHz ultrasound transducer through the temporal window. Mean flow velocity was evaluated 1 day before surgery. Patients were excluded from the study if the window for MCA blood flow evaluation was not found or the signal was not clear enough. According to the literature, about 5–37% of the patients had inadequate Doppler signals because of an insufficient acoustic temporal window or window failure during transcranial Doppler examination [15,16,17,18]. Hence, 15% of patients were rejected due to these reasons in our research. The Ml MCA stem usually lies at depths of 40–65 mm and is dependent on the size of the patient’s head [19]. It bifurcates into two divisions at a depth of 40–45 mm [19]. The monitoring was started by performing the technique at a depth of 50 mm; if the artery was not found or the waveform was not good enough, the depth was increased to 50–60 mm [19,20]. The normal spectral waveform shows a sharp systolic upstroke and stepwise deceleration with positive end-diastolic flow. The same operator performed all the measurements. The following evaluation was performed after anesthesia induction, before the beginning, the middle and the end of the CPB, and postsurgery in the ICU. Manual fixation of the probe was used. Monitoring was performed on both sides at a depth of 50–60 mm, depending on the patient’s constitution and curve quality. The normal values, according to the literature, were considered to be 30–80 cm/s [18].

### 2.4. Brain Biomarker

Blood samples for GFAP and neurofilaments heavy chain (Nf-H) were collected after anesthesia induction, 24 and 48 h after surgery. Both samples were analyzed by an enzyme-linked immunosorbent assay kit and designed for the quantitative measurements of GFAP and Nf-H in tissue and cell extracts. Measurements were made according to the manufacturer recommendations: GFAP—abcam, United Kingdom, Nf-H—antibodies-online, Germany. A signal was generated to the amount of bound analyte, and intensity was measured at 450 nm wavelength for both biomarkers. The lowest detection limits are defined as a mean-value of an analite-free sample plus three times the standard deviation and are the smallest clearly detectable concentration. According to the manufacturer’s recommendation, GFAP values in blood were 0.781 ng/mL and Nf-H 78.13–5000 pg/mL.

### 2.5. Statistical Analysis

The normality of data was assessed with Kolmogorov–Smirnov or Shapiro–Wilks tests. The study power (β) was calculated according to the incidence of dNCR. The dNCR was determined for 40% of patients. The estimated study power was 0.984, with a sample size of 101 patients and a 0.05 rate of type I error. The estimated size effect was 0.87, with a rate of type I error 0.05, type II error 0.2, the number of patients in the group I—41, in groups 0—60, and proportion 0.6. Normal distribution data were expressed as mean ± standard deviation (SD). Groups were compared by independent samples *t*-test. Non-normal distribution data were expressed as median (min, max). For nonparametric statistics, a Mann–Whitney U-test was performed for comparison between groups. The difference in frequency distribution between groups was determined by the chi-square test. Pearson correlation analysis was performed to determine the association between two variables (ACE-III test results and age, CO_2_, hematocrit, MCA BFV during bypass and others). 

The independent factors selection process was made with a univariate analysis of each variable. Any variable having a significant univariate test was selected as a candidate for logistic regression. Logistic regression was used to analyze how independent variables (age, MCA BFV during bypass, CO_2_, hematocrit) determine the probability of developing dNCR (dependent variable). dNCR was coded 0/1. The backward conditional method was chosen.

The statistical analysis was performed using IBM SPSS Statistics software (v. 23.0, Chicago, IL, USA). Statistical tests were two-sided, with *p* < 0.05 considered significant.

## 3. Results

### 3.1. Baseline Characteristics

In total, 101 of 140 patients completed ACE-III, MoCa, and CAM tests. They consisted of 33 females (32.70%) and 67 males (67.30%), mean age 69 (SD 9.10). 

dNCR was diagnosed if at least one test determined cognitive impairment, and it was identified for 41 (40.60%) patients. Delirium was diagnosed for 11 (10.90%) patients according to the CAM scale. 

According to the neurocognitive test results, patients were enrolled in two groups: patients without cognitive dysfunction after surgery were included in the first (non-dNCR) group and patients with delayed neurocognitive recovery were included in the second (dNCR) group. Groups were not differentiated according to sex, surgery type, comorbidities, duration of bypass and cross-clamping, ejection fraction, or bypass pump flow.

Demographic, preoperative, intraoperative, and postoperative characteristics are shown in Table 1.

### 3.2. Brain Biomarkers

GFAP changes are shown in Table 2. GFAP significantly increased 24 and 48 h after surgery in both groups. There was no statistically significant difference in GFAP concentration comparing non-dNCR and dNCR groups. There was no correlation found between neurocognitive test results or MCA BFV and GFAP concentrations in blood. 

Nf-H concentration changes in both groups are shown in Table 3. Nf-H elevation in blood was significant in both groups. There was no significant difference in Nf-H concentration comparing non-dNCR and dNCR groups. There was no correlation found between Nf-H concentration and neurocognitive test results or MCA BFV. 

### 3.3. MCA Blood Flow Velocity Changes

MCA BFV changes are shown in Table 4. 

BFV changed during surgery in the non-dNCR group. It was lowest during bypass, but this difference was not statistically significant. BFV notably decreased during bypass on the dNCR group (*p* = 0.006).

Comparisons between groups showed that BFV was lower during bypass and postsurgery in the dNCR group, compared to the non-dNCR group. 

The coefficient of variability interval was 1–30% (10.41%, SD 8.24) in the non-dNCR group and 1–36% (12.87% SD 9.58) in the dNCR group.

### 3.4. Pearson Correlation

Results of neurocognitive tests had a moderate positive correlation with CO_2_ concentration, hematocrit, and MCA BFV during bypass and moderate negative correlation with age (Table 5). 

### 3.5. Logistic Regression

Logistic regression revealed that age and MCA BFV were the most important factors for dNCR during the bypass. The probability of developing dNCR increases 1.22 times with every one year of increased age (*p* < 0.01). The probability of developing dNCR decreases 1.07 times with an increase of blood flow velocity within 1 cm/s during bypass (*p* = 0.02) (Table 6).

Logistic regression was used to analyze how independent variables (age, MCA BFV during bypass, CO_2_, hematocrit) determine the probability of developing dNCR (dependent variable). Method backward conditional was chosen. Only age and blood flow velocity during bypass have an influence on dNCR.

## 4. Discussion

Our research determined an elevation of brain biomarkers GFAP and Nf-H in both groups, but no correlation with dNCR. We found correlations between dNCR and age, hematocrit, CO_2_, and MCA BFV during bypass. MCA BFV severely decreased in the dNCR group during bypass. Regression analysis showed that only old age and reduction of MCA BFV during bypass increases the risk of dNCR.

An ideal biomarker should have high specificity for neural tissues and brain injury, rapid onset in blood serum, and a relationship between concentration and tissue injury [21]. The biomarker should also not respond to tissue damage caused by cardiac surgery [21]. Neurofilament heavy chain (Nf-H) and glial fibrillary acidic protein (GFAP) are among the biomarkers that correspond ideally to biomarker properties in cardiac surgery. GFAP has been reported to have a high sensitivity and specificity in traumatic brain injury, Alzheimer’s disease, and sepsis-associated encephalopathy [22,23]. NF-H had high specificity and sensitivity as a brain biomarker after cardiac arrest [24]. Nf-L was shown to be superior to other biomarkers for the prediction of recurrent stroke [25].

Neurofilaments (Nfs) are a group of proteins integrated into the neuronal and axonal cytoskeleton [11]. They are composed of three subunits: Nf-light, -medium and -heavy chains [11]. These polymers run along the length of axons while interacting with neighboring structures and other filaments [21]. Nf-H was selected for the ability to undergo posttranslational phosphorylation, which increases its resistance to breakdown by proteases and may facilitate its detection in blood serum [11].

Nf-H increased in both groups, but we did not find a correlation with dNCR. There is not much research with this biomarker in cardiac surgery, and the results are contradictory. Szwed K. compared several biomarkers suitable for cardiac surgery and did not find Nf-H elevation in patients with cognitive dysfunction [26]. Saller T. observed elevated neurofilament levels for patients with delirium after cardiac surgery [27]. It is already known that elevated levels of Nf are found after acute ischaemic stroke [6,28,29,30]. This biomarker could likely to be sensitive to cognitive impairment and brain injury, but more multicentered research is needed.

Nfs are new biomarkers with controversial results. One of the hypotheses is that preinterventional ischemia could mask any further alteration of pNf-H levels. Additionally, most of the investigations are of small sample size, and the ideal point for measurement is unknown [29]. Saller T (2019) observed that patients having high preoperative Nf-L levels (>40 pg/mL) experienced complications during CPB or delirium after surgery [27]. Another hypothesis is the incidence of increased Nfs reflects a higher vulnerability of the brain to exogenous triggers such as infection or CBP [27]. Opposite results have been obtained in different centers; the ideal cut-off point has not been determined, and there is little research on this topic. It is not possible to conclude the reliability of this biomarker in cardiac surgery. A multicenter study would help to unify the results and answer the question about the benefits of Nfs during cardiac surgery.

GFAP is a specific biomarker for astrocytes and found only in the central nervous system. For this reason, it is widely used as a biomarker for brain damage and in research. GFAP has been shown to be involved in astrocyte functions, which are important during regeneration, synaptic plasticity, and reactive gliosis [31]. In our research, GFAP increased in both groups, but there was no correlation with dNCR. Research has confirmed the GFAP association with dNCR. Vedovelli L.’s research showed impaired neurodevelopment is associated with an increase of GFAP plasma levels during cardiac surgery in infants [32]. Rappold T. demonstrated elevated GFAP levels for dNCR patients compared to noncardiac patients [33]. Gailiusas M. confirmed that increased GFAP levels are associated with early dNCR [34]. Easley R. B. determined impaired cerebrovascular autoregulation is associated with GFAP elevation [35]. However, other studies did not find a correlation between GFAP concentration and dNCR. Missler U. did not determine increased GFAP concentration for dNCR patients in cardiac surgery [36]. Sanchez-de-Toledo J.’s research demonstrated that serum biomarkers were not helpful in identifying neurological complications risk for children after cardiac surgery [37].

Research has confirmed that pre-existing brain vulnerability and neuronal damage are associated with dNCR [38,39,40]. GFAP and Nf cellular expression is reflective of intracellular molecules released by injured tissues during ongoing tissue necrosis [41,42,43]. Tissue injury could initiate neuroinflammation; it may also be initiated by CPB [44]. Some research has reported microglia activation and neuroinflammation as a consequence after cardiac surgery [45,46]. Any tissue inflammatory reaction is associated with the activation of the immune system; this can lead not only to recovery but also to further damage. For this reason, GFAP and Nf-H could be elevated for all patients, as this research showed. In addition, there is no evidence that these biomarkers have high specificity and sensitivity in diagnosing dNCR. Szwed K. examined the diagnostic potential of GFAP and Nf-H in cardiac surgery without bypass. Results revealed that these markers were not sensitive enough and not specific enough to diagnose delirium and dNCR [47]. This is, however, secondary to the results of this study, as we did not detect sufficient sensitivity and specificity in the diagnosis of dNCR. The most important risk factor for dNCR is age. Numerous research have confirmed the relationship between dNCR and age [1,48]. Older patients usually have structural brain changes caused by age, brain vessels atherosclerosis, or lower cognitive function reserves, which may lead to dNCR. In addition, research has revealed that elderly patients often have undiagnosed pre-existing brain disease [2]. Ito et al. investigated 449 elderly patients scheduled for cardiac surgery. Patients underwent cerebral magnetic resonance imaging and MR angiography preoperatively to assess cerebral infarctions and carotid and intracranial artery stenosis. Silent brain disease was found in 35.5% of patients [49]. Furthermore, elderly patients have many comorbidities, including structural changes in cardiovascular, pulmonary, and urogenital systems over the years, and this may manifest sensitivity to drugs, especially perioperatively [49]. This may also influence dNCR. 

Another important factor for dNCR is cerebral autoregulation and blood flow velocity. It is considered that cerebral hypoperfusion during surgery may lead to cognitive impairment. Our results and the results of many other investigations support this statement. Kosir G. determined hypoperfusion episodes in 9 of 15 patients in a pump–on group. MCA BFV was 15–25 cm/s in 5 patients and 5–15 cm/s in 4 patients. This study stated that blood flow velocity changes and pulsatility play an important role in postoperative neurologic outcomes [6]. Thudium M. indicated that relative cerebral hyperperfusion also disrupts cerebral autoregulation and might play a significant role in dNCR development [4]. Individual values were normalized to the pre-bypass baseline value, and MCA BFV > 100% was defined as cerebral hyperperfusion. The mean averaged MCA BFV was 90 (±21%) in the no-delirium group vs. 112 (±32%) in the delirium group (*p* < 0.05). It is interesting that a significant difference between the mean of MCA BFV during CPB (41.3 vs. 37.1 cm/s) was not found in delirium and no-delirium groups. Baseline MCA BFV was lower in patients who developed postoperative delirium (32.4 ± 12.0 cm/s vs. 46.1 ± 11.9 cm/s; *p* = 0.002). Benvenuti M. suggested that even preoperative BFV hypoperfusion may cause long term dNCR [13]. Nuttal Ga. noticed that MCA cerebral blood flow velocity changes during bypass as a percentage of baseline for all patients: pre-CPB BFV was 24 ± 6 cm/s, CPB start 27 ± 11 cm/s, CPB end 22 ± 10 cm/s, and post-CPB 32 ± 19 cm/s [50]. These results had been approved by this study. MCA BFV changed during surgery and the decrease of BFV during bypass was associated with dNCR.

BFV during bypass becomes nonpulsatile, which may be one of the risk factors of various complications [51,52]. Even short-term nonpulsatile flow conditions rapidly affect cerebral circulation [53]. Patients with low left ventricular ejection fraction receiving emergent VA-ECMO could potentially be vulnerable for cerebral insults, especially during hypothermic and hypocapnic states [53]. For this reason, it is very important to use multimodal monitoring, which may help to minimize the incidence of adverse neurologic reactions.

A validation study on the reproducibility of transcranial Doppler velocimetry was made by Maeda. CoV was determined at 6.7–19.5%, good enough to warrant the applicability of this method for the repeated measurements of the intracranial arterial blood flow velocity in future studies [54]. These measurements were performed on healthy individuals without any interventions. In this research were many influencing factors, from anesthesia to CPB, surgery, and medications that may change the result. Variability depends individually on each patient’s response to factors that were listed above. In this research, CoV was calculated for the same patient during different measurement times and showed that the values were distributed from low to high distribution. Since blood flow was affected by many factors, it is expected that the values could have been distributed more widely. 

Reduced variability (less than 6%) was determined for 24 patients (23.7%). These patients were statistically younger than the rest of the group (68 y (58; 75) vs. 69 y (59; 78); *p* = 0.45). This may have led to a better response of these patients’ blood vessels to various factors, and therefore, the blood flow velocity may have changed minimally. Increased variability (>20%) was determined for 4 patients (3.96%); all these patients suffered from dNCR and were associated with a less favorable outcome. 

A combined technique (blood biomarkers and BFV changes) was used to identify risk factors for dNCR. There are currently no studies combining these two methods in cardiac surgery. Our investigation determined that the most important factors for dNCR are age and blood flow velocity changes. Most patients undergoing cardiac surgery are elderly, so anesthesiologists should be especially careful. Monitoring anesthesia depth, brain oximetry, and blood flow velocity during surgery provide useful information and help to determine target doses of medicines, identify hypoperfusion or desaturation periods, and allow faster adjustment in treatment. Brain biomarkers GFAP and Nf-H are not helpful in identifying dNCR.

### Study Limitations

This study has several limitations. TCD was performed and BFV measured at the selected points of surgery. It would be more useful to monitor blood flow velocity continuously during all operations. This is a small sample of a one-center study. A multicentric study with a bigger sample size will provide more precise results. Additionally, there was no assessment of long-term neurocognitive disorders. Patients were discharged for rehabilitation or home, and we did not succeed in assessing mild neurocognitive disorders for most patients.

## 5. Conclusions

The most important factors in developing dNCR are older age and middle cerebral artery blood flow velocity decrease during bypass. The probability of developing dNCR increases 1.22 times with every one year of increased age (*p* < 0.01). The probability of developing dNCR decreases 1.07 times with an increase of blood flow velocity within 1cm/s during bypass (*p* 0.02) Neurofilaments heavy chain (Nf-H) and GFAP were not helpful in diagnosing dNCR, and we do not recommend their routine use.

## Figures and Tables

**Table 1:** Demographic preoperative, operative, and postoperative data of the patients.

	Non-dNCR Group	dNCR Group	*p* Value
Sex: male (n, %)	42 (70)	26 (63.40)	0.523
Age (years)	66.76(4.69) ^n^	72.00 (59; 77)	<0.01
Surgery type: CABG (n, %) Valve surgery (n, %) CABG + valve surgery (n, %)	40 (66.70) 15 (25.00) 5 (8.30)	26 (63.40) 10 (24.40) 5 (2.20)	0.815
Post myocardial infarction (n, %)	20 (33.30)	7 (7.10)	0.124
Atrial fibrillation (n, %)	9 (15)	9(22)	0.490
Duration of CPB (min)	97.07 (25.72) ^n^	92 (46; 173)	0.745
Duration of aortic cross-clamp (min)	53.12 (20.77) ^n^	49 (26; 138)	0.920
Haematocrit (Hct) CPB (%) ^n^	27.90 (1.46)	27.02 (1.47)	<0.01
CO_2_ CPB ^n^	42.29 (2.19)	41.01 (1.54)	0.01
CPB pump flow	3.96 (2.8; 9.94)	3.93 (0.44) ^n^	0.934
Ejection fraction (%)	50 (25; 60)	50 (20; 60)	0.724
Postoperative AF (n, %)	9 (15.00)	7 (17.10)	0.788

^n^ Data was normally distributed; they are presented as mean ± standard deviation. These groups were compared by independent samples *t*-test. Other data are presented as the median (min, max) or proportion, as appropriate. Mann–Whitney U test was performed for nonparametric statistics or when one of two variables distributed non-normally. The difference in frequency distribution between groups was determined by the chi-square test. AF—atrial fibrilation; CPB—cardiopulmony bypass; CABG—coronary artery bypass Graftin.

**Table 2:** Glial fibrillary acidic protein (GFAP) concentration in blood after anesthesia induction, 24 and 48 h after surgery.

	Non-dNCR Group	dNCR Group	*p*
GFAP induction (ng/mL)	0.008 (0.008; 0.43)	0.008 (0.0002; 0.54)	0.382
GFAP after 24 h (ng/mL)	0.13 (0.0005; 0.11)	0.009 (0.0001; 0.93)	0.342
GFAP after 48 h (ng/mL)	0.013 (0.0004; 0.11)	0.012 (0.000; 0.85)	0.273
*p*	<0.01	<0.01	

Data was distributed non-normally and presented as the median (min; max). Mann–Whitney U-test was performed for comparison between groups.

**Table 3:** Neurofilaments heavy chain (Nf-H) concentration after induction of anesthesia, 24 and 48 h after surgery in non-dNCR and dNCR groups.

	Non-dNCR Group	dNCR Group	*p*
Nf-H induction (pg/mL)	25.70 (2.88; 180)	41.16 (0.43; 180)	0.131
Nf-H after 24 h (pg/mL)	50.00 (2; 198)	54.20 (4.97; 180)	0.240
Nf- after 48 h (pg/mL)	45.49 (2.20; 198)	49.76 (0.72; 180)	0.597
*p*	<0.01	<0.01	

Data was distributed non-normally and presented as the median (min; max). Mann–Whitney U-test was performed for comparison between groups.

**Table 4:** Middle cerebral artery blood flow velocity changes during surgery.

	Non-dNCR Group	dNCR Group	*p*-Value
MCA BFV before surgery (cm/s)	45.92 (13.17)	41.83 (12.82)	0.125
MCA BFV after induction (cm/s)	44.96 (12.31)	40.99 (11.91)	0.110
MCA BFV bypass (cm/s)	43.40 (9.56)	37.13 (7.70)	0.001
MCA BFV post-surgery (cm/s)	47.76 (12.01)	40.54 (11.21)	0.003

Data were normally distributed for these variables; they are presented as mean ± standard deviation. Groups were compared by independent samples *t*-test.

**Table 5:** Correlations between the Adenbrooke test (ACE-III) neurocognitive test result and CO_2_ concentration, hematocrit, and MCA BFV during bypass and age.

	Pearson Correlation	*p*
CO_2_ concentration during bypass (mmHg)	0.40	<0.01
Hematocrit during bypass (%)	0.42	<0.01
MCA BFV bypass (cm/s)	0.41	<0.01
Age (years)	−0.53	<0.01

MCA—middle cerebral artery; BFV—blood flow velocity.

**Table 6:** Logistic regression analysis results.

	B	SE	Wald	Df	Sig.	Exp(B)	95% C.I. for EXP (B) Lower/Upper
Age	0.197	0.05	13.90	1	<0.01	1.22	1.098/1.351
MCA BFV bypass	−0.71	0.03	5.42	1	0.020	0.93	0.877/0.989
